# Insights into the post-transcriptional regulation of the mitochondrial electron transport chain

**DOI:** 10.1042/BST20160100

**Published:** 2016-10-19

**Authors:** Tamara M. Sirey, Chris P. Ponting

**Affiliations:** 1 Institute of Genetics and Molecular Medicine, MRC Human Genetics Unit, University of Edinburgh, Edinburgh, U.K.; 2 Department of Physiology, Anatomy and Genetics, University of Oxford, Oxford, U.K.

**Keywords:** eukaryotic gene expression, metabolic regulation, microRNA, mitochondrial respiration, RNA-binding proteins

## Abstract

The regulation of the mitochondrial electron transport chain is central to the control of cellular homeostasis. There are significant gaps in our understanding of how the expression of the mitochondrial and nuclear genome-encoded components of the electron transport chain are co-ordinated, and how the assembly of the protein complexes that constitute the electron transport chain are regulated. Furthermore, the role post-transcriptional gene regulation may play in modulating these processes needs to be clarified. This review summarizes the current knowledge regarding the post-transcriptional gene regulation of the electron transport chain and highlights how noncoding RNAs may contribute significantly both to complex electron transport chain regulatory networks and to mitochondrial dysfunction.

## Introduction

The control of metabolic homeostasis is central to maintaining the physiological function and health of an organism. A key player in the maintenance of cellular homeostasis is the semi-autonomous mitochondria, which produce ∼95% of cellular ATP through the coupling of the electron transport chain (ETC) to oxidative phosphorylation (OXPHOS), by a proton electrochemical gradient across the mitochondrial inner membrane. Mitochondria also provide the environment for other metabolic pathways, such as the Kreb's cycle, β-oxidation and the urea cycle, and they also regulate Ca^2+^ homeostasis and play a key role in cellular apoptosis. Metabolic homeostasis is co-ordinated by a combination of key transcription factors and post-transcriptional regulatory mechanisms, including noncoding RNAs that combine to form intricate regulatory control networks. However, much remains to be understood about the role post-transcriptional processes play in the maintenance and regulation of the ETC and how they provide a further insight to the complexities underlying metabolic homeostasis.

Post-transcriptional gene regulation (PTGR) can broadly be defined as the control of gene expression at the level of RNA transcript abundance and includes aspects of RNA biology, such as transcript stability/RNA turnover, binding of the RNAs by RNA-binding proteins (RBPs) and post-transcriptional regulation by microRNAs (miRNAs). It is known that ETC transcripts range from being moderately- to highly expressed and are relatively stable [1], but little is known regarding the role of regulatory RNAs or RBPs in the PTGR of the ETC. This review aims to provide an overview of the current evidence for post-transcriptional regulation of the mitochondrial ETC and to discuss the role PTGR may play in diseases that exhibit mitochondrial dysfunction.

The chemiosmotic coupling of the ETC to OXPHOS requires the activities of four multisubunit enzyme complexes (complex I [CI], NADH-ubiquinone oxidoreductase; complex II [CII], succinate-quinone oxidoreductase; complex III [CIII], cytochrome *bc*_1_ complex; complex IV [CIV], cytochrome *c* oxidase), and ATP synthase (complex V [CV]) as the site of OXPHOS, numerous assembly proteins and two electron carriers (ubiquinone and cytochrome *c*). Together, the complexes and electron carriers comprise the ETC that transfers electrons from NADH (at CI) and FADH_2_ (at CII) through a series of redox reactions to molecular oxygen as a final electron acceptor (at CIV). In doing so, protons are pumped across the inner mitochondrial membrane to create a proton electrochemical gradient that is required for ATP synthase to phosphorylate ADP to produce ATP (OXPHOS), therefore providing energy for cellular processes. In mammals, the five enzyme complexes comprise ∼100 separate protein subunits and are unique in the sense that their protein components are sourced from two separate genomes — the mitochondrial genome and the nuclear genome — which necessitate the co-ordinated (post)-transcriptional regulation of genes from both genomes. Since the mitochondrial genome only codes for 13 of these protein subunits (7 CI, 1 CIII, 3 CIV and 2 CV), the majority of ETC subunit transcripts are encoded by the nuclear genome, translated in the cytoplasm and their proteins imported into the mitochondria. The efficient function of the ETC therefore requires complex layers of regulation to co-ordinate the expression of the protein-coding subunits, including a combination of transcriptional co-ordination, sub-cytoplasmic localization of translation and the intricate assembly of the ETC enzyme complexes.

## Post-transcriptional regulation of the ETC via miRNAs

miRNAs are small (21–23 nucleotide) noncoding RNAs that post-transcriptionally regulate target genes in the cytoplasm through the activity of the multicomponent RNA-induced silencing complex (RISC). This occurs by the miRNA binding via a seed region in the mature miRNA, to a miRNA recognition element in a target sequence, with canonical binding being mostly targeted to the 3′-UTR. This interaction of the miRNA with its target RNA is recognized by the Argonaute 2 component of RISC and either suppresses protein production and/or initiates mRNA degradation [2].

**Figure 1.**
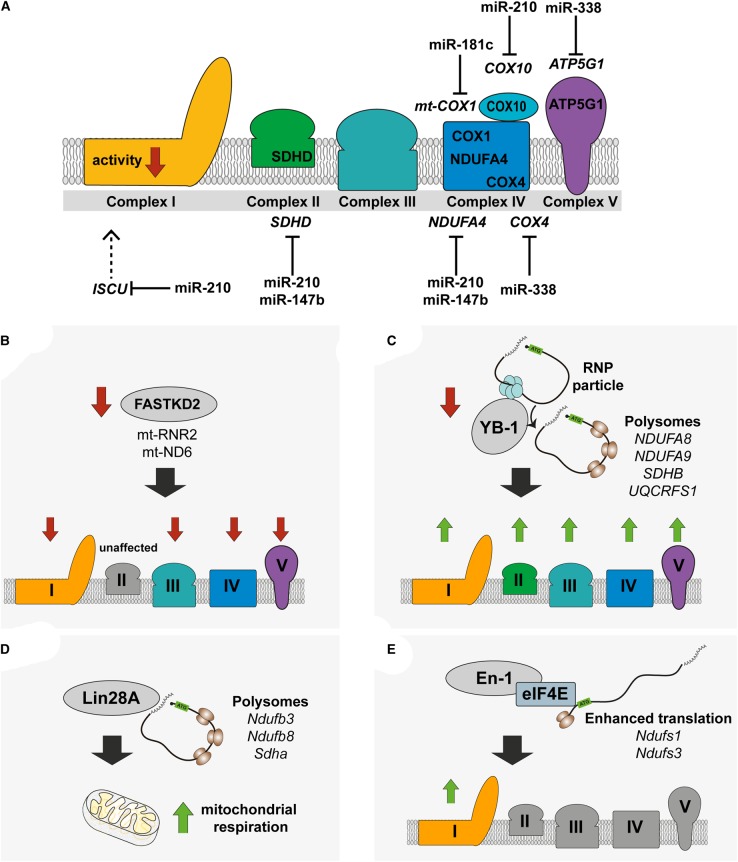
Summary of the post-transcriptional gene regulation mechanisms known to regulate ETC transcript abundance. (**A**) Schematic representation of the ETC complexes indicating which transcripts have known miRNA-mediated regulation that has a downstream biochemical effect. (**B**) Down-regulation of the RBP *FASTKD2* results in a decrease in activity of all mitochondrial complexes that contain mitochondrially encoded subunits. (**C**) Down-regulation of the RBP *YB-1* results in release of ETC transcripts from ribonucleoproteins (RNPs) and subsequent recruitment to the polysomes for translation, leading to increase in catalytic activity of all complexes. (**D**) Lin28A binds to and enhances the translation of some ETC transcripts leading to an overall increase in mitochondrial respiration. (**E**) The homeobox transcription factor En-1, through an interaction with the eukaryotic translation initiation factor eIF4E, specifically enhances the translation of two mitochondrial CI transcripts, leading to an increase in CI enzymatic activity.

miRNAs can confer robustness to gene expression networks by suppressing ‘noise’ (for a review and examples, see refs [3–5]), and by establishing gene expression thresholds, which can help maintain homeostasis. At present, miRNAs are known to participate in the regulation of various metabolic pathways, including insulin signaling, glucose homeostasis and lipid homeostasis [6,7]. Nevertheless, except for a few specific examples (discussed below), little is known about the magnitude of the role that miRNAs may play in the regulation of ETC transcripts (Figure 1A).

Many studies have reported interactions of miRNAs with ETC transcripts in different biological contexts. The brain-specific miRNA, miR-338, has been shown to locally regulate cytochrome *c* oxidase IV (a complex IV subunit) transcript abundance in the axons of sympathetic neurons [8], thereby regulating axonal respiration. A subsequent study identified the transcript ATP5G1 as an additional target of miR-338 in axons [9], suggesting that miR-338 may be co-ordinately regulating the availability of multiple ETC subunit transcripts in this system.

The miRNA miR-210, which is induced under hypoxic conditions, has been demonstrated to act as part of the metabolic switch in the hypoxic response [10]. In human pulmonary artery cell lines, miR-210 was initially shown to target transcripts encoding the iron–sulphur cluster assembly enzymes *ISCU1*/*2* [10], which is a mitochondrially localized scaffold protein required for the maturation of [2Fe–2S] and [4Fe–4S] proteins [11]. By targeting *ISCU1*/*2*, the induction of miR-210 decreases the activity of Fe–S-containing enzymes such as the Kreb's cycle enzyme aconitase [10] and mitochondrial complex I [10,12]. The CII subunit *SDHD* has also been validated as an miR-210 target [13–15], as has the CIV assembly factor *COX10* [12] and *NDUFA4* [14–16], which has previously been reassigned to CIV [17]. These multiple miR-210 targets allow the co-ordinated regulation of mitochondrial respiration in response to hypoxia first by regulating the enzyme ISCU, which has downstream effects on the Fe–S-containing proteins of both the Kreb's cycle and the ETC and, secondly, by directly targeting ETC subunit transcripts and assembly factors to further decrease ETC activity. In addition, in human placentas presenting with preeclampsia, a two-fold increase in miR-210 is associated with decreased amounts of translated complexes CI and CIV and a decrease in the activity of CIII [18], implying an even broader role of miR-210 in the regulation of the ETC. These various targets of miR-210 are suggestive of a hypoxia-induced post-transcriptional regulatory network, which acts to co-ordinately down-regulate key ETC enzymes involved in the hypoxic response.

There is evidence of overlapping regulatory functions between miRNAs that share target genes. miR-210 and miR-147b are induced by hypoxia and in inflammation, respectively, and promote comparable cellular effects in terms of cell migration, proliferation and apoptosis [15]. These miRNAs share a minimal six-base seed region and both directly interact with *SDHD* and *NDUFA4* transcripts. This miRNA functional redundancy, where different external stimuli can trigger the expression of different miRNAs, acts as a ‘switch’ that leads to a common stress-related response characterized by overlapping miRNA-target genes [15].

Intriguingly, both pre- and mature-miRNAs (termed mitomiRs) have been found localized to the mitochondria, and miRNAs have been found to be present in the mitochondria isolated from rat liver [19], mouse liver [20], 143-B cells [21], myoblasts [22], HeLa cells [23] and HEK293 cells [23]. There is little consensus, however, regarding the functionality of these miRNAs within the mitochondria, and it is important to note that only one of these studies [22] used hybridization techniques to demonstrate miRNA localization to the mitochondria; therefore, artifacts due to the fractionation techniques utilized cannot be precluded. The mitochondria have been proposed to act as an miRNA reservoir owing to some mitomiRs being predicted not to target the mitochondrial genome or nuclear-encoded mitochondrial protein transcripts, but instead, transcript-encoding proteins involved in apoptosis and cell proliferation and differentiation [19]. It is important to note that as of yet the targets of mitomiRs have not yet been extensively validated experimentally. Nevertheless, one miRNA, miR-181c, has been shown to be functional in the mitochondria of rat cardiac myocytes where it localizes within the mitochondria and translationally regulates mt-COX1 [24]. In addition, as there is evidence that Argonaute proteins localize to the mitochondria [24,25], suggesting that the proteins required for miRNA-mediated gene silencing may be present within the mitochondria. Furthermore, there is some evidence that the mitochondrial genome itself generates noncoding RNAs [21,26], although specific mitochondrial targets remain to be validated experimentally. A deep-sequencing approach identified small RNAs generated from either the mouse or the human mitochondrial genome ranging in size from 12 to 137 nucleotides [27]. In contrast with miRNA-mediated repression, these mitochondrial genome-encoded small RNAs (mitosRNAs) appear to enhance the expression of their mitochondrial host genes [27]. It remains to be determined how extensive is the mitosRNA regulation of mitochondrial gene expression, but it could represent a further component of PTGR.

It should be noted that the miRNA pool do not bind just mRNAs, but potentially other noncoding transcripts such as expressed pseudogenes, long noncoding RNAs (RNAs >200 nucleotides in length with no-coding capacity) and circular RNAs. These transcripts can thus act as miRNA decoys, or competitive endogenous RNAs (ceRNAs), by binding miRNAs that would otherwise bind specific target mRNAs; these mRNAs are thus derepressed [28–30]. There is potential for the miRNA-mediated regulation of ETC subunits to be buffered by a miRNA:mRNA:lncRNA network that maintains cellular homeostasis.

## Post-transcriptional regulation of the ETC via binding of ETC transcripts to RNA-binding proteins

Both nuclear- and mitochondrially encoded ETC transcripts can also bind to and be sequestered by RNA-binding proteins (RBPs). These are not only numerous and diverse, but they also have various cellular functions ranging from RNA modification in the nucleus (splicing, polyadenylation and 5′ capping), mRNA export, mRNA turnover, mRNA localization and translation [31]. Previously, the FASTK family of FAS-activated serine/threonine kinases has been identified as non-canonical RNA RBPs implicated in mitochondrial physiology [32–34]. FASTKD4 (FAS-activated serine/threonine kinase D4) has been shown to mediate the turnover of a subset of mitochondrially encoded transcripts [35], whereas FASTKD2 (FAS-activated serine/threonine kinase D2) acts as an RBP that interacts with the mitochondrially encoded transcripts 16S ribosomal RNA (*RNR2*) and the complex I subunit *ND6* [36]. Deletion of *FASTKD2* results in aberrant processing and expression of both *RNR2* and *ND6* with a subsequent decrease in the activity of all respiratory complexes, with the exception of CII [36] (Figure 1B). FASTKD2-mediated post-transcriptional regulation of these genes is a critical cellular process because homozygous nonsense mutations in the *FASTKD2* gene are associated with mitochondrial encephalomyopathy [33].

Other RBPs have been shown to promote the expression of ETC subunits. In HeLa cells, it has been demonstrated that YB-1 (Y-box-binding protein-1) regulates the translation of a subset of nuclear-encoded ETC subunits by recruiting mRNAs from inactive ribonucleoprotein particles to active polysomes [37]. The ability of YB-1 to act as a translational activator depends on the amount of YB-1 bound to the target mRNA. For example, after siRNA-mediated YB-1 depletion, the protein expression levels of CI (NDUFA9 and NDUFA8), CII (SDHB) and CIII (UQCRFS1) subunits were increased by approximately 50%, with a concomitant increase in respiratory chain activity [37] (Figure 1C), suggesting that YB-1 is an important mediating factor for modulating the translation of ETC subunits.

In mice, the RBP LIN28a is a repressor of let-7 miRNA biogenesis, but it also regulates mRNA translation independently [38]. In mouse embryonic fibroblasts and mouse pinnae, LIN28a binds to, and enhances the translation of, transcripts encoding the CI subunits *Ndufb3* and *Ndufb8*, and the CII subunit *Sdha*, in addition to glycolytic and Krebs cycle transcripts [38]. This Lin28a-mediated translational up-regulation results in an increase in mitochondrial respiration, and is part of the Lin28-mediated reprogramming of metabolism that enhances tissue repair [38] (Figure 1D).

Although the homeobox proteins are well known as transcription factors, some also function as translational regulators by interacting with the eukaryotic translation initiation factor eIF4E [39]. Exogenous application of the homeobox protein *engrailed-1* (*En-1*) enhances the translation of the core mitochondrial subunits *Ndufs1* and *Ndufs3*, which lead to a 20% increase in the activity of CI (Figure 1E). In turn, this protects mouse midbrain dopaminergic neurons against the CI inhibitor used to model Parkinson's disease, 1-methyl-4-phenyl-1,2,3,6-tetrahydropyridine [40]. It also doubles striatal dopamine concentrations, an effect that is dependent on the translation of *Ndufs1* [40].

Together, this experimental evidence suggests that miRNAs and RBPs post-transcriptionally regulate nuclear-encoded mitochondrial subunits and contribute centrally to the regulation and homeostatic control of energy metabolism.

## High-throughput detection of miRNAs and ETC targets

Genome scale technologies have previously been implemented to identify differentially expressed miRNAs and/or miRNA targets. miRNA microarrays were used to identify miRNAs that are differentially regulated during mouse aging in combination with global proteomic profiling strategy to identify differentially expressed proteins [41]. This strategy identified 27 miRNAs that were up-regulated with a concomitant decrease in the expression of 10 ETC proteins (CIII: UQCR2, UQCRB and UQCRFS1; CIV: COX5A, COX5B and COX7A2; ATP synthase: ATP5B, ATP5F1, ATP5H and ATP5O) in aging mice. A similar approach using miRNA and mRNA microarrays was taken to identify differentially expressed miRNAs and target genes in neural precursors derived from human umbilical cord mesenchymal stem cells [42]. The present study identified miR-34a as an miRNA involved in neurogenesis, and demonstrated that expression of miR-34a results in the down-regulation of at least eight ETC subunit transcripts (CI: *NDUFA3*, *NDUFB2*, *NDUFB7* and *NDUFS6*; CIII: *UQCR*; CIV: *NDUFA4*; CV: *ATP5F1* and *ATP5G3*).

Many studies are now combining high-throughput sequencing with different approaches that investigate RNA interactions with proteins, specifically Argonaute proteins (e.g. HITS-CLIP, PAR-CLIP and CLASH), to identify miRNA-target interactions (for a review of RNA–protein interaction technology, see ref. [43]). These studies are investigating miRNA:target interactions at a transcriptome-wide scale and therefore provide an excellent resource of potential miRNA:mRNA interactions that can be mined to identify targets for further investigation. Table 1 illustrates examples of datasets where potentially novel ETC:miRNA interactions have been identified.

**Table 1:** Example of potentially novel miRNA interactions with ETC transcripts identified from high-throughput screening datasets of RNA:protein interactions Datasets were downloaded from miRTarBase [48].

Species/cell line	Technique	Number of miRNA:ETC transcript interactions	Non-redundant number of ETC transcripts identified	Non-redundant number of miRNAs
Human HEK293 cells [44]	CLASH	295	74	104
Human 293S and HeLa cells [45]	HITS-CLIP	422	27	307
Human HIV-1-infected C8166 T cells or TZM-bl epithelial cells [46]	PAR-CLIP	249	14	231
Human HEK293 cells [47]	PAR-CLIP	229	18	210

These high-throughput techniques provide a broad transcriptome-wide overview of potential miRNA:target interactions. Nevertheless, confidence in the validity of the proposed interactions requires subsequent experimental validation.

## Does PTGR of the ETC play a central role in mitochondrial dysfunction?

While much is understood about the biochemical mechanisms underlying oxidative phosphorylation, in situations of mitochondrial dysfunction, where mitochondria fail to generate appropriate amounts of ATP in response to energy demands, little is known about the underlying causes. Mitochondrial dysfunction is an important pathophysiological feature of many apparently disparate diseases, including neurodegenerative disorders such as Parkinson's and Alzheimer's diseases [49–51], mental health disorders [52,53], type II diabetes [54] and heart disease [55]. Inherited mutations in protein-coding genes are often not sufficient to explain the prevalence of sporadic cases of these diseases, and increased susceptibility to complex neurodegenerative and neuropsychological disorders are likely to be the product of multiple mutations. Given the etiological complexity of these diseases, it is possible that mutations in PTGR networks regulating the ETC may contribute to the observed pathologies.

## Concluding statement

The extent to which PTGR influences the activity of the ETC has yet to be fully elucidated. However, there is clear experimental evidence that both miRNAs and RBPs have the potential to play significant post-transcriptional regulatory roles in different disease contexts. If we add to this post-transcriptional network other noncoding RNAs, such as the ceRNAs, we begin to envisage a complex post-transcriptional network that can respond rapidly to stimuli to preserve homeostasis. Importantly, a better understanding of these networks could help to determine what roles they may play in diseases that manifest defects in energy metabolism and could help identify much needed new drug targets for treating mitochondrial dysfunction.

## Abbreviations

ceRNAs, competitive endogenous RNAs; CI, complex I; CII, complex II; CIII, complex III; CIV, complex IV; CV, complex V; En-1, engrailed-1; ETC, electron transport chain; FASTKD2, FAS-activated serine/threonine kinase D2; FASTKD4, FAS-activated serine/threonine kinase D4; miRNA, microRNA; mitosRNAs, mitochondrial genome-encoded small RNAs; mitomiRs, mature-miRNAs; OXPHOS, oxidative phosphorylation; PTGR, posttranscriptional gene regulation; RBPs, RNA-binding proteins; RISC, RNA-induced silencing complex; RNPs, ribonucleoproteins; YB-1, Y-box-binding protein-1.

## Competing Interests

The Authors declare that there are no competing interests associated with the manuscript.
